# Isolated Torticollis May Present as an Atypical Presentation of Meningitis

**DOI:** 10.1155/2012/193543

**Published:** 2012-10-21

**Authors:** Roger Chirurgi, Samrina Kahlon

**Affiliations:** ^1^Emergency Department, New York Medical College, Metropolitan Hospital Center, New York, NY 10029, USA; ^2^New York Medical College, Metropolitan Hospital Center, New York, NY 10029, USA

## Abstract

*Background*. Bacterial meningitis is a life-threatening medical emergency that requires urgent diagnosis and treatment. Diagnosis is infrequently missed if the patient presents with the classic symptoms of fever, headache, rash, nuchal rigidity, or Kernig or Brudzinski sign. However, it may be less obvious in neonates, elderly, or immunocompromised patients. Meningitis which presents as isolated torticollis, without any other signs or symptoms, is exceedingly rare. *Objective*. To identify an abnormal presentation of meningitis in an adult immunocompromised patient. *Case Report*. We present a case of an adult diabetic male who presented multiple times to the ED with complaint of isolated torticollis, who ultimately was diagnosed with bacterial meningitis. *Conclusion*. We propose that in the absence of sufficient explanation for acute painful torticollis in an immunocompromised adult patient, further evaluation, possibly including a lumbar puncture may be warranted.

## 1. Introduction

Bacterial meningitis is not uncommonly seen in the Emergency Department (ED). There are nearly 3,000–8,000 cases of meningitis every year in the United States [1]. It commonly presents with headache, photophobia, fever, rash, nuchal rigidity, or Kernig or Brudzinski sign. Isolated torticollis, without any other signs or symptoms, is exceedingly rare. A review of the literature resulted in only two reported cases, both in pediatric patients [2, 3]. We present here a case of an adult diabetic male who presented multiple times to the ED with complaint of isolated torticollis, who ultimately was diagnosed with bacterial meningitis. 

## 2. Case Report

A 59-year-old male presented to the ED with a chief complaint of neck pain for five days. The patient reported that he was in his usual state of health and awoke with “a stiff neck” five days prior. He reported that two days later he went to see his private physician who prescribed him methocarbamol without relief. He subsequently presented for the first time to our ED. He reported involuntary contractions of the neck muscles, causing his head to be held to the left. He denied headache, photophobia, nausea, vomiting, fever, upper extremity weakness or numbness, stridor, dysphagia, drooling, odynophagia, or respiratory symptoms. His past medical history was significant for hypertension, diabetes (controlled with oral medication), and osteoarthritis. On social history he denied smoking, alcohol, or illicit drug use. On physical exam, his vital signs were HR 70, RR 18, BP 131/81, and temperature 36.4°C (97.6°F) orally. He was noted to be alert and oriented, cooperative, and in obvious mild discomfort from pain. Neurologic exam revealed clear speech, normal cranial nerve exam. Pupils were pinpoint with poor reaction to light, without photophobia. HEENT exam revealed normal intact TM with no evidence of otitis. No nasal discharge, no pharyngeal erythema, and no tonsilar inflammation was noted. His head was held in left rotation. The neck exam was negative for midline vertebral tenderness but positive for left paraspinal tenderness. Decreased range of motion of the neck (left to right and up to down) was noted as secondary to pain. Extremity examination was noted to have full strength in the upper extremities bilaterally with intact triceps reflexes, decreased range of motion in the lower extremities (which patient reports is longstanding and secondary to osteoarthritis), and positive right lower extremity ulcer with dressing in place. The patient was treated with ketorolac 60 mg IM and had a CT of his head and neck which were read by the radiologist as “normal aerated sinuses, no sign of mass effect or shift. No sign of an acute bleed, minimal atrophy.” The CT of the neck was read as “degenerative changes but no sign of a discrete fracture, the prevertebral soft tissues appeared intact. There is some straightening. A significant compression was not seen.” The patient reported significant relief of pain with ketorolac and was subsequently discharged with a prescription for Tylenol number 3 and followup with PMD. The patient returned to the ED four days later, with chief complaint of continued neck pain. He continues to deny headache, fever, nausea, or vomiting. He continued to be afebrile with an unchanged examination. Again he was treated with ketorolac and discharged home this time with acetaminophen/hydrocodone, cyclobenzaprine, and ibuprofen. 

The patient returned for a third time to the ED six days later (now day 15) for a near syncopal episode. He reported feeling dizzy after getting up from bed, falling to the floor, and being unable to get up off the floor. He reported that the cyclobenzaprine made him dizzy. A neighbor found him and called EMS. At the time of presentation, the patient indicated he had had a stiff neck for two weeks. He denied chest pain, palpitations, headache, vertigo, fever, chills, nausea, or vomiting. On examination he was noted to be tachycardic to 120 beats per minute. His physical exam was again noted to be unchanged from his previous two presentations. His working diagnosis was noted to be “presyncope, possibly side effect of medication, rule out ACS, neck pain.” EKG was noted to be normal rhythm, nonacute. His admission CBC was noted to have significant leukocytosis of 34,500 with 92% neutrophils. He subsequently received a lumbar puncture which was significant for a cerebrospinal WBC count of 1,310; 94 Segs, 2 Bands, 2 Monos, RBCs 30; no organisms seen, bacterial antigen STREP Group B positive. The patient was admitted and received intravenous antibiotics, did well, and was ultimately discharged from hospital after day ten.

## 3. Discussion 

Nuchal rigidity is a well-described clinical manifestation of meningitis. It refers to stiffness of the neck that prevents flexion. Neck stiffness is a protective reflex that shortens the spinal axis restricting movement and immobilizing the irritated tissue, causing a spasm of the exterior muscles [4]. In some cases this may be more severe leading to neck pain, torticollis and opisthotonus [5].

Torticollis refers to presentation of the neck in a twisted or bent position. The name derives from the Latin for *torti,* which means twisted and *collis,* which means neck. Torticollis manifests in involuntary contractions of the neck muscles, leading to abnormal postures and movements of the head. Torticollis can be either congenital or acquired. The differential diagnosis includes trauma, tumors, infection (including lymphadenitis following upper respiratory infections, cervical adenitis, and retropharyngeal abscess), cervical disc disease, and drug induced. We will limit our discussion to the acquired torticollis [6–8]. “It often results from an inflammatory process that irritates the cervical muscles, nerves, or vertebrae. Posturing of the head occurs with unilateral spasm of the sternocleidomastoid muscle such that the child will position the head with the occiput rotated to the affected side and the chin rotated to the contralateral side” [9].

In regards to presentation, in two large case studies of patients with meningitis the prevalent symptoms were headache (50%), lethargy (50%), fever (61%), GI symptoms (30%), convulsions (40%), respiratory distress (47%), jaundice (28%), irritability (32%), and neck pain (28%) in most patients [10, 11]. Isolated torticollis, without any other signs or symptoms, is exceedingly rare. A review of the literature resulted in only two reported cases, both in pediatric patients. Our patient presented to the Emergency Department on three separate occasions with torticollis without any of the typical signs or symptoms of meningitis. The pain improved promptly on treatment with NSAIDs, and the muscle spasms improved with muscle relaxants and benzodiazepines. We propose that in the absence of well-recognized presenting signs and symptoms of meningitis, the presence of unexplained torticollis, in an immunocompromised patient, a lumbar puncture should be considered to completely rule out the diagnose of bacterial meningitis.
